# Enhanced Compression Properties of Open-Cell Foams Reinforced with Shear-Thickening Fluids and Shear-Stiffening Polymers

**DOI:** 10.3390/polym17091218

**Published:** 2025-04-29

**Authors:** Jian Li, Yaoguang Zhou, Mohammad Rauf Sheikhi, Selim Gürgen

**Affiliations:** 1 The State Key Laboratory of Heavy-Duty and Express High-Power Electric Locomotive, Central South University, Changsha 410075, China; jianli1@csu.edu.cn (J.L.); 224212076@csu.edu.cn (Y.Z.); mohammadraufsheikhi@csu.edu.cn (M.R.S.); 2 Key Laboratory of Traffic Safety on Track of Ministry of Education, School of Traffic & Transportation Engineering, Central South University, Changsha 410075, China; 3 National & Local Joint Engineering Research Center of Safety Technology for Rail Vehicle, Central South University, Changsha 410075, China; 4 Department of Aeronautical Engineering, Eskişehir Osmangazi University, Eskişehir 26040, Turkey

**Keywords:** open-cell foams, shear-stiffening polymers (SSPs), shear-thickening fluids (STFs), compressive properties, rheological characterization

## Abstract

Open-cell PU foams have a wide range of industrial applications due to their excellent cushioning, impact protection, packaging, thermal insulation, and sound reduction benefits. The foams absorb impact energy while deforming under compressing and are ideal for applications with severe and repeated loading conditions. Enhancing and improving their compressive durability is a vital area of ongoing research. We investigated the effect of incorporating shear-stiffening polymers (SSPs) and shear-thickening fluids (STFs) on the compression properties of open-cell foams. Rheological properties of STFs and SSPs prepared for incorporation into the foams confirmed the shear-thickening and shear-stiffening characteristics. Quasi-static compression tests performed at different speeds (6, 60, 120, 180, and 240 mm/s), as well as load-unload compression tests (6 and 24 mm/s), showed that the SSP-filled foam exhibited the most pronounced improvement in the elastic, plateau, and densification regions compared to the neat foam. While the STF-filled foam also improved performance over the neat foam, its advantages over the SSP-filled foam were less pronounced. The performance of the SSP-filled foam improved with increasing compression speeds, while the performance of the STF-filled foam remained relatively stable between 60 and 240 mm/s of load-unload tests. Post-test compression evaluations showed that neat and STF-filled foams quickly regained their original shape, while SSP-filled foams required more time before recovery. This research shows that combining SSP and STF smart materials with open-cell foams substantially improves their compressive performance, especially at high compression rates and load-unloading scenarios, increasing their functional life.

## 1. Introduction

Multiple engineering and industrial applications use open-cell foams as a key material for cushioning and packaging requirements alongside thermal insulation and sound damping. These foams, typically made from polyurethane (PU) materials, have distinctive mechanical properties that allow them to absorb applied energy while deforming under compressive forces [1]. Open-cell foams exhibit excellent compressibility characteristics in severe and repeated loading conditions. To achieve optimal performance, these structures must maintain compression over time without suffering structural damage or permanent collapse [2,3,4]. Various techniques have been developed to improve the compression properties of open-cell foams and extend their service life. Experimental results show that impregnating open-cell foams with shear-stiffening polymers or gels (SSPs or SSGs) and shear-thickening fluids (STFs) can enhance their impact resistance and improve their compressive properties [5].

STFs are non-Newtonian-based fluids that exhibit high viscosity when suddenly applied forces provide energy dissipation upon impact [6,7]. On the other hand, SSPs act as materials that increase stiffness after experiencing mechanical tension, which helps improve the foam’s resistance to deformation [8,9]. Many studies have demonstrated the usability of SSPs and STFs in anti-impact [10,11,12,13,14], energy-absorbing [15], vibration-damping [16,17,18], and polishing [19,20] applications by integrating them into various engineering structures, suggesting their high potential due to their unique shear-thickening and stiffening properties. Adding STF and SSP to foams substantially alters their overall mechanical properties and increases impact resistance by improving energy-absorption capability as the material becomes more viscous under shear forces. The foam benefits from better and more efficient energy dissipation, which helps protect it from damage [21]. SSPs increase foam integrity and reduce compression through stiffening effects that maintain the shape and operational capacity of the foam during compression [22].

STFs and SSPs have recently become the subject of research to improve open-cell foams’ compression and impact resistance. Livermore et al. [23] studied STF-filled PU foams, which showed rising compressive stress measurements because of shear rate differences in the fluid contained within the foam structure. Liu et al. [24] confirmed optimal compression energies in STF capsule-filled PU composite foams when using PU65/30. Warren et al. [25] studied hypervelocity impact tests on metallic foam core sandwich panels containing STF and discovered that the infused panels outperformed ones containing Newtonian polyethylene glycol liquid. Wu et al. [26] fabricated sandwich composites using polyurethane grid-sealed STF core material to achieve better static compression and dynamic impact properties. Incorporating STF in foam structures increased their impact-resistance properties, while higher STF volume content produced a stronger compression modulus and enhanced impact strength. Tu et al. [27] presented a composite foam by combining shear-thickening gel (STG) and ethylene–vinyl acetate (EVA) foam, which improved the elasticity and viscosity of the modified material compared to neat EVA foam. In another study [28], they subjected the composite foam composed of STG and EVA to ballistic tests, and the results of the tests and simulations showed that the deformation caused by head impact on the human body was notably reduced by using EVA modified with STG as a buffer material. By conducting quasi-static and in situ compression tests, they also showed that the combination of STG and EVA improved their compression properties in the elastic, plateau, and densification regions [21]. Liu et al. [29] investigated the low-velocity impact and compressive properties of STG/PUF composites, along with modifications in their chemical structure. The results showed that the addition of STG enhanced the foam’s mechanical properties and the composites’ physical entanglement, causing the molecular parts of STG and PU to interpenetrate and form a more stable structure. They also presented a multifunctional composite with energy absorption and a high strain rate by adding STG and thermal expansion microspheres as reinforcements to PU [30].

Existing research on open-cell foam composites containing STFs and SSPs focuses mainly on axial and quasi-static compressive tests. At the same time, compression rate effects, particularly cyclic loading compression, are poorly understood. This research fills this gap through an extensive experimental analysis of STF- and SSP-reinforced foams under different compression rates and load-unloading conditions. This study underscored the practical importance of understanding the rate-dependent behavior and performance under cyclic loading for applications where these materials are subjected to demanding and repeated compressive forces (e.g., sports protective gear, automotive crash-absorbing structures). Our study aims to gain a deeper understanding of the compressive properties of SSP- and STF-based foams and gain insights into their optimization and potential application in situations requiring high compressive strength.

## 2. Materials and Methods

### 2.1. Materials

Foamtech (Shenzhen, China) provided the polyurethane (PU) foam used in this study. The foam had a density of 50 ± 1 kg/m^3^; and a porosity of 85%. Fumed silica (SiO_2_) particles, averaging 20 μm in size, were sourced from Shanghai Bowei Applied Materials Technology (Shanghai, China). Analytical chemical reagents, including polyethylene glycol (PEG, 400 g/mol), absolute ethanol, and acetone, were supplied by Merck (Darmstadt, Germany), which also provided boric acid and silicone oil (1000 cSt). Benzoyl peroxide (BPO) was obtained from Thermo Scientific Chemicals (Waltham, MA, USA).

### 2.2. Specimen Preparation and Structural Characterization

**Figure 1:**
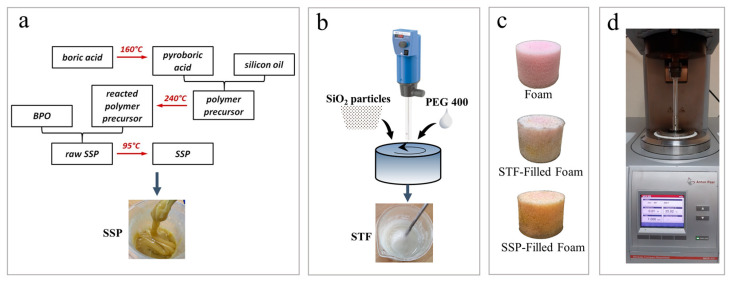
The fabrication process of (**a**) SSP, (**b**) STF, (**c**) final smart foams, and (**d**) rheometer.

The synthesis of STF involved incorporating SiO_2_ powder into the PEG matrix, followed by a 1.5 h homogenization using a high-speed homogenizer (T25, IKA). The percentage contribution of SiO_2_ to PEG was 58%, and ethanol was used for ease of addition. For the SSG fabrication, boric acid was first heated to 160 °C for two hours to prepare pyroboric acid. Then, 2 g of prepared pyroboric acid was mixed with 15 g of silicone oil and 1 mL of absolute ethanol. This blend was reacted at 240 °C for 8 h to create the polymer precursor. After this process, 4 wt% BPO was added to the prepared polymer precursor and vulcanized at 95 °C for two hours. STFs and SSGs were dissolved in an ethanol bath and combined with the foams. The ratio of ethanol to STF was 1:2, while the ratio of ethanol to SSG was 1:3. Cylindrical specimens, with a diameter of 40 mm and a thickness of 30 mm, prepared for compression tests, were immersed in these solutions. To address potential concerns about ethanol treatment, it is important to note that ethanol’s rapid evaporation at room temperature would likely result in minimal residual effects on the foam samples. After complete immersion, the sponges were dried at 60 °C for 12 h to evaporate the ethanol. The fabrication process of SSP and STF is illustrated in Figure 1a,b. Microstructural evolution in the smart foams was examined using scanning electron microscope (SEM) images taken with a Hitachi SU8230 system. A PerkinElmer Spectrum Two system was also utilized to analyze the sponge’s chemical structures via Fourier-transform infrared (FTIR) spectroscopy. The spectra were acquired from the wavenumber range of 4000 to 500 cm^−1^ with a resolution of 4 cm^−1^. To further understand the rheological behavior of the fabricated STF and SSG, these materials were analyzed using an Anton Paar MCR 302 rheometer (Figure 1d). For the STF, the shear rate was gradually increased from 0 to 1000 s^−1^ to generate the viscosity flow curve, employing a 25 mm parallel plate configuration. Due to the viscoelastic nature of the SSG, oscillatory testing was conducted. These tests were performed at a shear strain of 1%, with the shear frequency ranging from 0.1 to 1000 Hz. The storage modulus (G′) was used to assess the elastic component of the SSG, indicating its stiffness and energy stored during deformation. Conversely, the loss modulus (G′) assessed the viscous properties of the SSG, reflecting the energy dissipated as heat during deformation. The rheological analysis emphasizes the qualitative differences between the STF and SSP, emphasizing their contrasting behaviors rather than precise quantitative values.

### 2.3. Compression Testing

**Figure 2:**
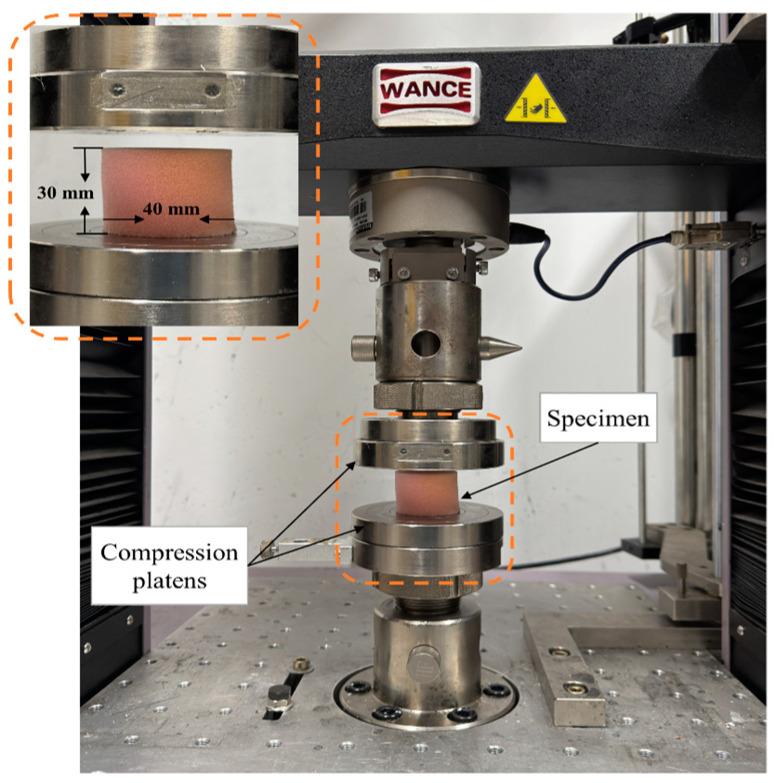
The setup is used for open-cell smart foam compression testing.

The samples’ compressive strength was measured at rates of 6, 60, 120, 180, and 240 mm/min using a universal testing machine (TSE-B, Wance Testing Machine Co., Ltd., Shenzhen, China), as shown in Figure 2. The samples were compressed to 80% of the sample thickness for standard compression tests. Furthermore, to better analyze the compression properties of the design foams, the load-unloading test on the samples through four cycles with 6 and 240 mm/min compression rates was conducted. First, we compressed the samples to a thickness of 10 mm and then released them at the same compression rate (cycle 0–10 mm). The samples were then compressed to 15, 20, and 24 mm thicknesses and then decompressed to their original state at the same compression rate (cycles 0–15, 0–20, and 0–24). Each experiment was repeated three times to ensure statistical reliability, and the average values were reported with standard deviations. The tests followed ASTM D1621 standards [31] for compressive properties of rigid cellular materials. We also calculated the energy dissipation during these load-unloading compression tests on the designed foams. To accomplish this, we used the trapezoidal rule to analyze the force–displacement curves from each cycle. The area under these curves shows how much energy was dissipated during compression and released during decompression. The difference between the areas for loading and unloading represents the energy dissipation, which reflects the foam’s internal friction and hysteresis during deformation. The sample thickness was 30 mm, and the diameter of the samples was 40 mm. The experimental design is given in Table 1.

## 3. Results and Discussion

### 3.1. Rheological Properties of STF and SSP

**Figure 3:**
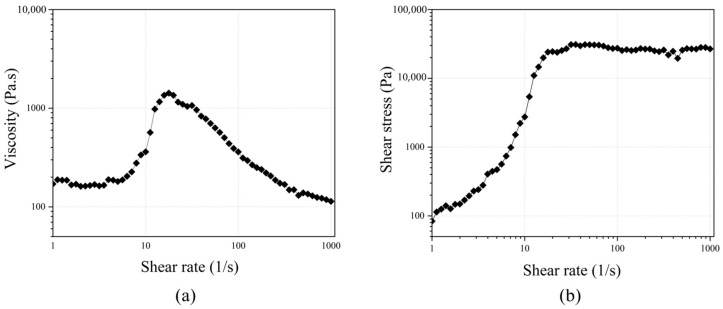
Rheological analysis of STF: (**a**) viscosity as a function of shear rate, (**b**) shear stress as a function of shear rate.

The rheological characteristics of our manufactured STF are displayed in Figure 3. In Figure 3a, the initial viscosity of 170 Pa.s suggests a relatively low resistance to flow at low shear rates, akin to the behavior of Newtonian fluids. When the shear rate rises and reaches a critical point (5.625 1/s), the viscosity increases significantly, peaking at 1422 Pa.s. The shift indicates shear-thickening behavior, where the STF changes from a fluid to a more solid-like state, enhancing its resistance to deformation under applied stress. Past this critical point, the STF’s viscosity decreases, which is indicative of the shear-thinning phase, where the fluid structure deteriorates under sustained shear. As shown in Figure 3b, the initial shear stress (83.8 Pa) quickly rises with increasing shear rates, peaking at approximately 30,500 Pa. This nonlinear increase confirms the shear-thickening property of STF, which demonstrates significant stiffness under higher shear stresses. These rheological properties emphasize STF’s potential for applications requiring adaptive mechanical properties, such as protective gear, damping systems, and other impact-resistant technologies. The dual behavior of STF, exhibiting both shear-thickening and shear-thinning, is advantageous for effectively managing energy dissipation and absorption under variable shear conditions.

**Figure 4:**
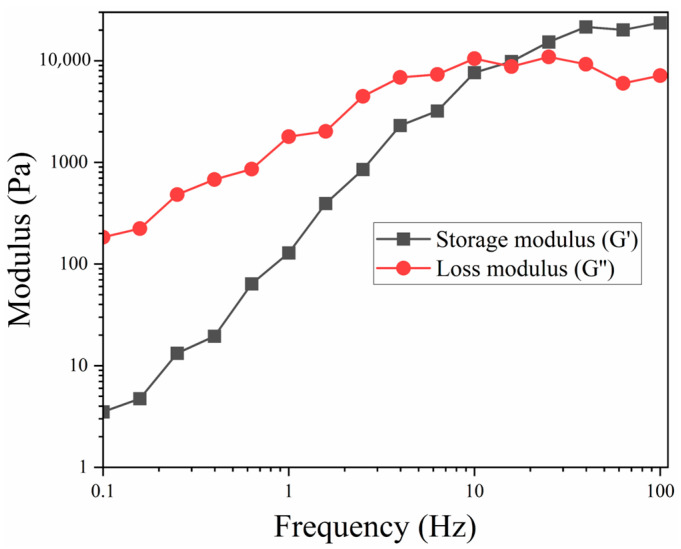
Storage and loss moduli properties of SSP.

Figure 4 illustrates the rheological behavior of the SSP. At a frequency of 0.1 Hz, the storage modulus starts at 3.2 Pa, indicating the polymer’s initial stiffness and its ability to store elastic energy at low shear rates. As the shear rate increases, the storage modulus rises sharply, reaching 23,600 Pa at 100 Hz, which suggests that the SSP becomes stiffer and more elastic as the frequency and shear rate increase, thus improving its ability to store elastic energy. Additionally, the loss modulus, which begins at 183.8 Pa at 0.1 Hz, signifies the polymer’s viscous behavior and its energy dissipation properties. Loss modulus also increases with frequency, peaking at 7140 Pa at 100 Hz, although this rise is less pronounced than that of the storage modulus. These variations in loss modulus suggest increased energy dissipation and internal friction within the polymer at elevated frequencies. The intersection of storage modulus and loss modulus at about 10 Hz indicates the critical transition frequency, where the material behavior shifts from mainly viscous to more elastic. This transition frequency is vital for grasping the dynamic response of the SSP. Overall, the increase in both moduli with frequency showcases the SSP’s adaptive mechanical properties, essential for its performance under different shear conditions. These findings provide valuable insights into the balance between elastic energy storage and energy dissipation in SSP, contributing to a deeper understanding of its intricate rheological behavior.

### 3.2. Microstructural Properties of Smart Foams

**Figure 5:**
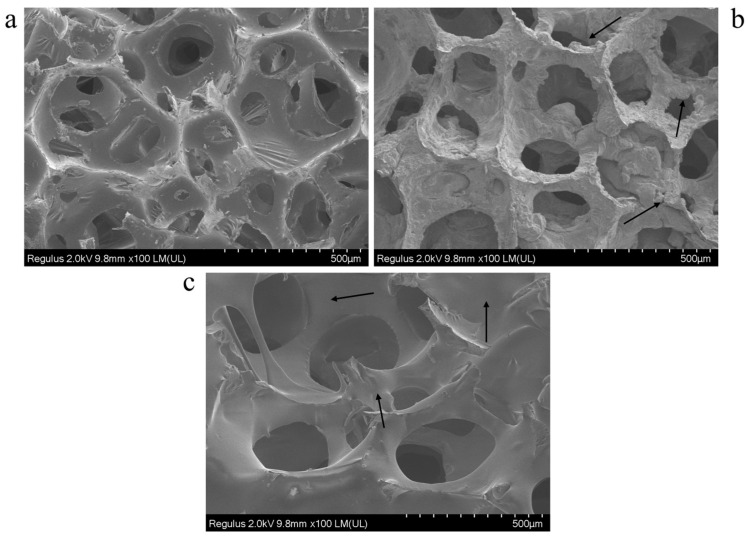
SEM images of (**a**) foam, (**b**) STF-filled foam, and (**c**) SSP-filled foam (magnification: 100×).

As illustrated in Figure 5, SEM imaging shows clear distinctions between the designed foam samples. A typical open-cell structure in a foam sample consists of interconnected voids and uniform cell-wall surfaces. As indicated by the arrows in Figure 5b, STF-filled foam exhibits thicker cellular walls with rough surfaces because STF integrates into the foam structure. The integration process exhibits inconsistent characteristics because the structure shows points of varying thicknesses alongside regions of roughness. The microstructure of SSP-filled foam shows improved uniformity among its cellular elements. As shown in Figure 5, arrows indicate uniform coating and film formation on cell walls, showcasing the SSP material’s smooth and continuous coverage. The SSP-filled foam exhibits porosity with a better-defined structure and reduced irregularities compared to the STF-filled foam because of a more precise reinforcement method.

### 3.3. Anti-Compression Performance of Smart Foams

#### 3.3.1. Quasi-Static Compressive Behavior at Different Compression Speeds

**Figure 6:**
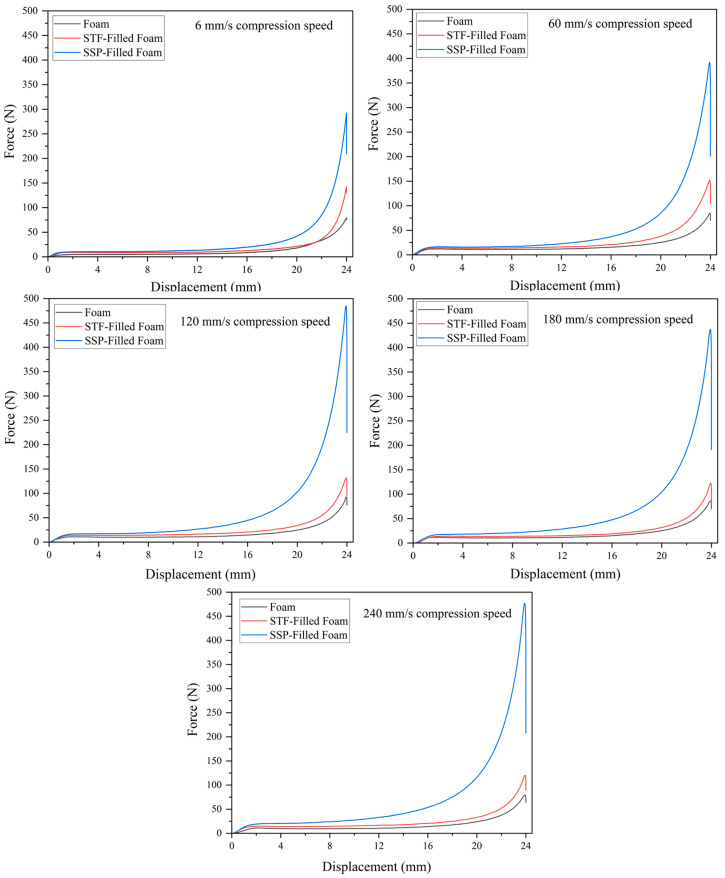
Compression behavior of designed smart foams at different compression speeds.

Figure 6 shows the compression behavior of the designed smart foams at different compression speeds. Results show that SSP-filled foams surpass all mechanical features in all deformation areas (elastic, plateau, and densification) compared with neat and STF-filled foams at all compression rates (6, 60, 120, 180, and 240 mm/s). STF-filled samples demonstrated moderate performance gains over neat foam, especially in the densification region. The enhanced performance of the SSP-filled foam is due to the characteristics of SSPs, which show shear-stiffening behavior. When a compressive force acts, the SSPs are integrated within the foam structure and change from flexible to rigid, enhancing resistance to deformation [28]. This change enables the foam to absorb and dissipate energy during the deformation regions, increasing strength and durability for all deformation regions. In particular, in the plateau region, where the foam is deformed considerably at low stresses, the shear-stiffening effect of the SSPs runs to the site. In this region, the stiffer SSPs enhance the foam’s cellular structure and mitigate collapse, ensuring the structural integrity of the foam. The interactions among foam cells in the plateau region of the foam are critical to the observed improvements. Under shear forces, the SSP reinforces the foam’s cell walls and decreases the likelihood of buckling or collapse.

**Figure 7:**
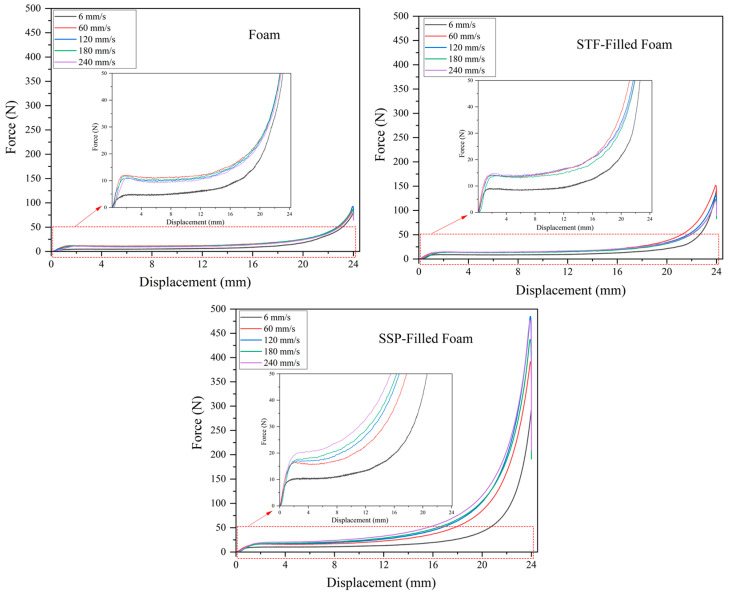
Compression behavior of designed smart foams with increasing compression speeds. The red dotted area indicates the region shown in the magnified inset.

Figure 7 shows the compression behavior of designed smart foams with increasing compression speeds. In the case of the neat foam, the compression behaviors do not change noticeably for the different compression speeds, suggesting that the mechanical properties of the conventional foam sample are not noticeably dependent on the compression rate. The improvement in compression performance with the STF-filled sample appears marginal, particularly in regions of densification, where higher compression rates typically lead to improved performance to an extent. In comparison, the SSP-filled sample shows the most pronounced improvement in the elastic, plateau, and densification deformation regions as the compression rate increases. This continuous improvement indicates that the SSP-filled foam is more elastic to the variation of the velocity of compression, which can be related to the intrinsic structure of SSPs. With increasing compression rates, the applied deformation forces act faster, boosting the shear-stiffening effect. The stiffening properties of SSPs in the plateau region control the foam cells from collapsing while maintaining their structural integrity and distributing stress throughout the foam structure. The SSPs exhibit enhanced stiffening behavior that boosts foam structural support as compression rates increase, thus improving the strength and longevity of the material. In conclusion, the superior performance of SSP-filled foam at higher compression speeds can be attributed to the coupling effect between polymer-chain entanglement dynamics and loading rate. At higher rates, the SSP polymer chains experience forced conformational changes that increase entanglement density and crosslinking efficiency, resulting in greater stiffening effects [30]. While previous research has demonstrated the strain-rate sensitivity of similar composite materials for tensile properties [30] and axial compressing [23], our findings indicate a more substantial enhancement in compressive strength compared to values reported in the existing literature, making it particularly suitable for applications where compressive strength is a critical factor.

#### 3.3.2. Load-Unloading Behavior of Smart Foams

**Figure 8:**
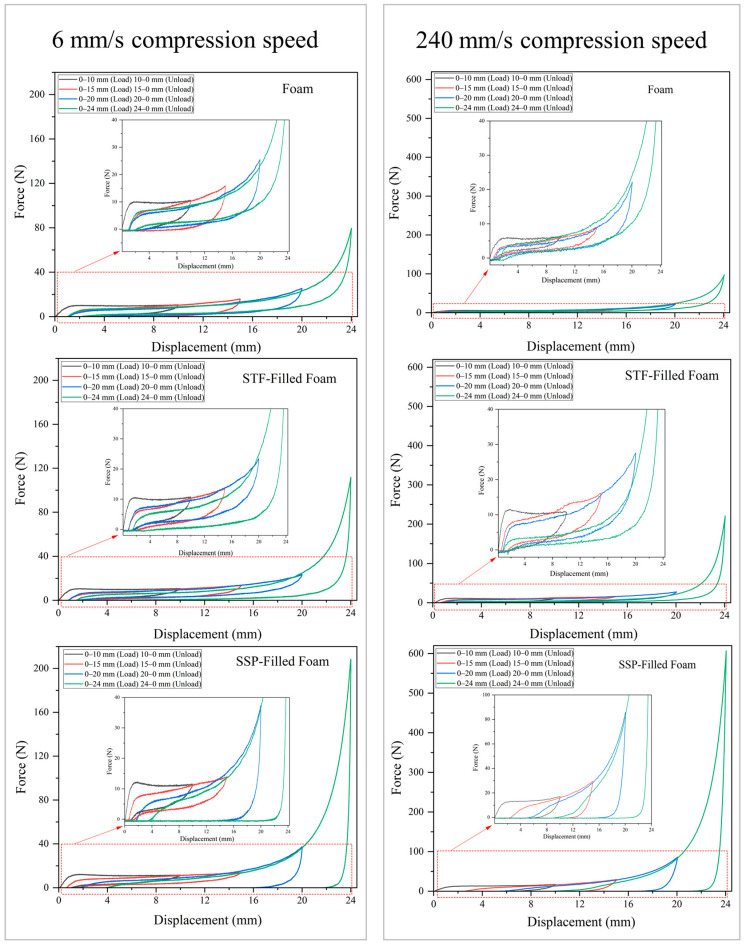
Load-unloading compression behavior of designed smart foams at 6 and 24 mm/s compression speed. The red dotted areas indicate the region shown in the magnified inset.

As shown in Figure 8, load-unloading compression tests were performed on the designed specimens at 6 and 240 mm/s, revealing impressive aspects of their mechanical behavior. At a compression rate of 6 mm/s, the neat foam exhibits typical open-cell foam behavior, with the elastic and plateau regions reaching approximately 10 N in the initial load-unloading ranges and a peak compressive force of approximately 75 N. The STF-filled foam showed moderate improvements in the elastic and plateau regions of approximately 11 N and a maximum compressive force of 100 N in the high-compression range. The SSP-filled foam with an elastic and plateau zone of about 13 N in the initial load-unloading ranges and 180 N in the densification zone of the load-unloading region showed the most pronounced improvement. At a compression rate of 240 mm/s, the mechanical properties of the conventional foam remained constant, with an elastic zone of about 7 N and a peak force of 75 N. The STF-filled sample showed improved compressive properties with elastic and plateau regions of about 12 N. The SSP-filled foam with elastic and plateau zones of about 15 N and a peak compressive force of 580 N in the 0–24, 24–0 load-unloading range showed the most noticeable improvement.

Due to the specific shear-thickening and shear-stiffening mechanisms of the addition of STFs and SSPs in open-cell foams, their compressive performance is substantially enhanced when applied to a load. STFs thicken when applied to a load, ultimately leading to higher energy absorption and resistance to foam deformation. This feature is an additional advantage in the compression process, where the STF-filled foam showed an improvement in strength compared to the neat foam. Although the STF-filled samples performed better than the neat foams, the overall improvement in their compressive properties was slight, and the most pronounced improvement in the compressive strength of the foam occurred in the densification region. On the other hand, SSPs exhibit shear-stiffening behavior, which means their stiffness increases when subjected to a load. In our case study, this behavior led to an increase in the compressive properties of the foam over several deformation ranges. The SSP-filled samples’ elastic, plateau, and maximum compressive strength zones substantially enhanced performance, leading to better performance. These results confirm the ability of SSPs to improve the mechanical strength and stability of foams under high stresses.

The trend of improving the mechanical strength of the foam was more pronounced at higher compression rates and when increasing the rate from 6 mm/s to 240 mm/s in the SSP sample. This means that the reinforcement of open-cell foams with SSP is better for applications with dynamic loading and places exposed to instantaneous or high compression rates. Conversely, the improvements of the STF-filled foam were less sensitive to the compression rate, which allows them to be used in applications that require regular improvements of the foam’s compressibility during application. These results highlight the need for careful selection of adding SSPs and STFs when tuning the compressive properties of open-cell foams to the application requirements.

**Figure 9:**
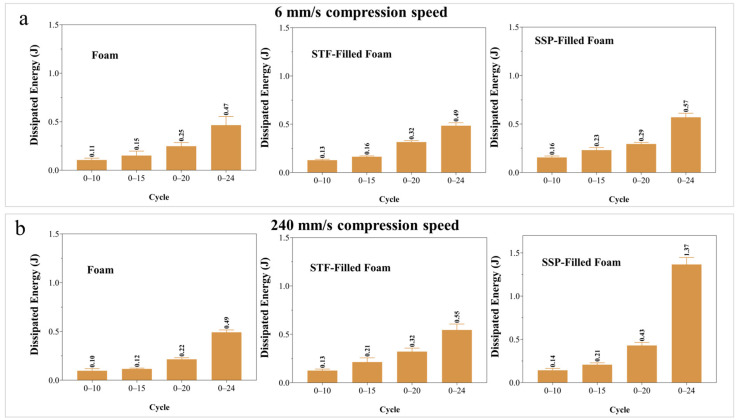
Dissipated energy of designed smart foams during load-unloading cycles: (**a**) 6 mm/s compression speed, (**b**) 240 mm/s compression speed.

The dissipated energy results shown in Figure 9 provide valuable insight into the enhanced energy-absorption properties of open-cell foams modified with STFs and SSPs. These results are based on load-unloading compressing data, highlighting SSP-filled foams’ increased efficiency at higher compressing velocities. By examining the load-unloading at 6 mm/s, the neat foam has the least dissipated energy in all cycles, and 0.473 J (0 to 25 mm) is the maximum dissipation energy. The STF-filled foam is better but only slightly at 0–10, 0–15, and 0–20 cycles. This slight increase is caused by the STF’s sharp ST behavior, which helps absorb loads but incurs no heavy drop in foam structure and behavior. Compared to other samples, SSP-filled foam absorbs much energy in 0–10 and 0–15 cycles, reaching 0.561 J in the 0–25 mm cycle. This improvement is due to how the SSP resists changes in behavior and can absorb energy better. At the 240 mm/s compression rate, the samples’ differences become more substantial. The STF-filled foam performs better at all cycles than the foam sample due to the STF’s behavior, which will provide superimposed thickness as the strain rates rise. SSP-filled foam performs surprisingly well at this comparatively slow speed. It achieves a maximum dissipated energy of 1.374 J, doubling the results of STF-filled foam and almost tripling neat foam. This notable increase is because the SSPs stiffen dynamically at high strain rates, which helps absorb and dissipate much more applied energy.

### 3.4. Reversibility Performance of Designed Smart Foams

**Figure 10:**
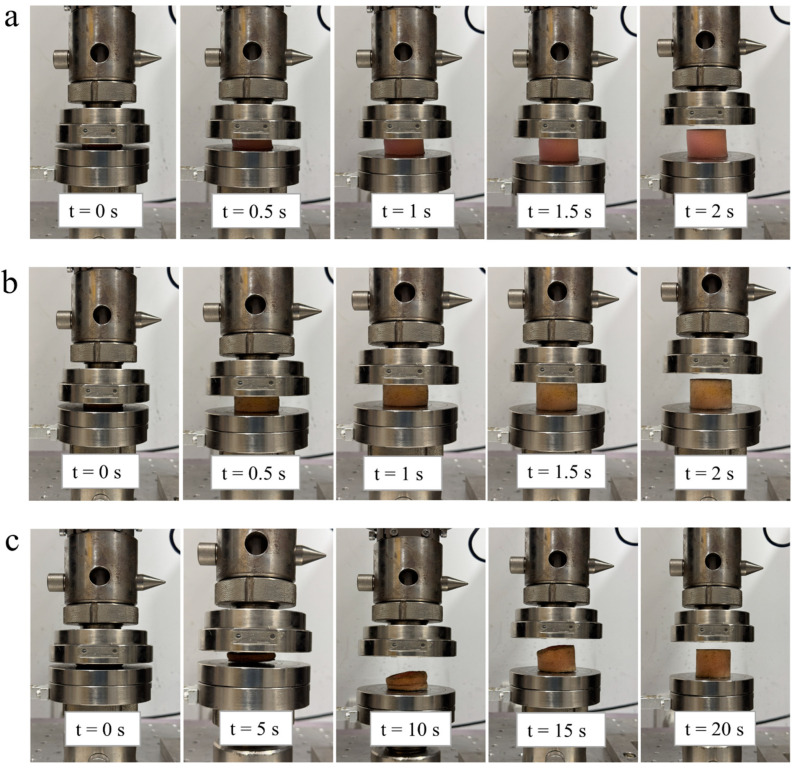
The ability to restore the original shape of (**a**) foam, (**b**) STF-filled foam, and (**c**) SSP-filled foam samples.

After carrying out compression tests on the foam samples, they were found to be recoverable, which highlights their differences in viscoelastic properties. Figure 10 illustrates the smart foams’ capability to regain their original shape. The neat foam and STF-filled foam quickly returned to their original shapes, whereas the SSP-filled foam took almost 20 s to recover. This is clearly due to its material properties and the SSP-network interaction with foam. The quick return of the neat foam is typical of an open-cell PU foam, as it does have an elastic return due to having less internal resistance. Likewise, the STF-filled foam also showed an immediate recovery, as the STF basically dissipates energy during compression while not substantially impeding the elastic recovery of the foam. STFs become thicker when they are under stress, but return to their original state immediately when the stress is off. This trait allows the STF-filled foam to absorb energy well when loaded but maintain a recovery profile similar to that of neat foam. The viscoelastic behavior of SSPs gives the foam the ability to act as a shear-stiffening network when stress is applied, reducing the time it takes for the structure and cellular network to recover. This stiff network does not immediately relax upon unloading. Instead, it has to take time for the disentanglement of the polymer chains inside the SSP and the dissipation of the internal stresses, causing slower recovery. This phenomenon represents the built-in trade-off between enhanced mechanical properties and reduced reversibility. The delayed recovery of SSP-filled foams suggests that these types of foams, like slow-rebound or memory foams, are more suitable for applications where energy absorption and structural integrity under high-speed loading are more important than their immediate recovery. Slow-rebound foams are a popular cushioning material because of their excellent shape memory, powerful energy absorption, shock absorption, and shock resistance [29]. In contrast, STF-filled foams are ideal for applications that require energy dissipation and rapid recovery. The designed foams’ recovery results demonstrate the importance of selecting a foam that can be optimized for a specific set of performance requirements with viscoelastic properties and recovery dynamics.

## 4. Conclusions

This study investigated the effects of incorporating STF and SSP smart materials on the compressive properties of open-cell PU foams. At first, the rheological behavior of STF and SSP materials prepared for incorporation into the foams was investigated, and results indicated that the fabricated STF and SSP exhibited shear-thickening and shear-stiffening properties. Then, quasi-static compression tests performed at different displacement rates (6, 60, 120, 180, and 240 mm/s) revealed that SSP-based foams exhibited better compression performance in all deformation regions (elastic, plateau, and densification). For example, their strength increased to 30 N in the plateau and 480 N in densification regions at a compression rate of 240 mm/s, nearly threefold in the plateau and sixfold in the densification region compared to neat foam. Although STF-based foams improved, especially in the densification region, their performance was lower than that of SSP-based foams. Furthermore, load-unload compression tests demonstrated SSP-based foams’ advantages, indicating superior energy absorption and mechanical strength. For instance, their strength increased almost three times in the plateau (15 N) and six times in the densification region (600 N) compared to the neat foam in the 0–24 load-unload cycle. Also, SSP-based foams had a slower reversibility time due to the increased density and viscosity of the SSP fillers, taking approximately 20 s to return to their original shape, while neat and STF-based foams recovered almost immediately. The findings of this study indicated that incorporating SSPs into open-cell foams substantially improves their compressive properties, especially at higher compression rates.

Based on our findings, STF-based foams are suitable for applications requiring continuous energy absorption at varying compression rates and rapid recovery after compression, such as packaging for sensitive electronic and medical equipment. SSP-based foams’ advantages extend beyond sensitive electronic and medical applications to areas requiring gradual stiffening responses, such as sports protective gear (e.g., helmet liners, body padding), automotive crash-absorbing structures, and military protective equipment. SSP- and STF-based smart foam limitations can be attributed to manufacturing challenges, aging, and environmental factors such as temperature and humidity, which should be addressed in future studies. Prospective studies should also optimize SSP crosslinking density and network structure to improve recovery kinetics while maintaining desirable stiffening properties.

## Figures and Tables

**Table 1:** Design of experiments for designed smart foams.

Specimen	Details
Foam	Neat foam
STF-filled foam	STF-impregnated composite sponge
SSP-filled foam	SSP-impregnated composite sponge

## Data Availability

The datasets presented in this article are not readily available because the data are part of an ongoing study.
